# Creation of an Implementation Blueprint for the National Emergency Airway Registry for Pediatric Emergency Medicine (NEAR4PEM) Pre-Intubation Checklist

**DOI:** 10.21203/rs.3.rs-8491286/v1

**Published:** 2026-01-20

**Authors:** Robyn Wing, Ariana M. Albanese, Monica M Prieto, Emily Greenwald, Ilana Harwayne-Gidansky, Joshua Nagler, Michael P. Goldman, Joshua Ray Tanzer, Kelsey Miller, Natalie Napolitano, Akira Nishisaki

**Affiliations:** Brown University; Brown University; University of Pennsylvania, Children’s Hospital of Philadelphia; Duke University School of Medicine; Albany Medical College; Boston Children’s Hospital; Yale School of Medicine; Brown University; Boston Children’s Hospital; Children’s Hospital of Philadelphia; Alpert Medical School of Brown University and Rhode Island Hospital, Hasbro Children’s Hospital

**Keywords:** modified conjoint analysis, pediatric intubation, checklist

## Abstract

**Background:**

The National Emergency Airway Registry for Pediatric Emergency Medicine (NEAR4PEM) developed an evidence-based pre-intubation checklist, however its successful integration to clinical practice in the Pediatric Emergency Department (PED) requires attention to implementation. Given the complex conditions influencing checklist use, it is essential to work with key informants to understand multilevel determinants and identify the most effective strategies for implementation. The objective of this study was to systematically identify barriers to checklist adoption and to prioritize and detail targeted strategies as an implementation blueprint to support successful checklist integration into clinical practice.

**Methods:**

NEAR4PEM recruited Airway Champion (AC) teams composed of physicians, nurses, pharmacists, and respiratory therapists at each PED. Our methodology consisted of a five-step modified conjoint analysis. In Step 1, a mixed-methods formative evaluation was conducted, utilizing focus groups and surveys for identification of barriers and facilitators to checklist implementation. In Step 2, key informants prioritized the identified barriers according to feasibility and impact quantitatively via survey. In Step 3, the prioritized barriers were matched with implementation strategies from a published compilation (Expert Recommendations for Implementation Change, ERIC) via virtual facilitated sessions. In Step 4, these strategies were ranked for feasibility and impact by Advisory Board (AB) members. In step 5, the AB detailed the prioritized implementation strategies in an implementation blueprint.

**Results:**

In Step 1, ACs from 13 sites completed 45 surveys, which, together with focus groups, identified 16 unique barriers. For Step 2, these key informants prioritized 6 barriers of high impact and high feasibility. For Step 3, an implementation science team assisted ACs with selection of 24 ERIC strategies. In Steps 4 and 5, the AB prioritized 19 ERIC strategies and incorporated them into an implementation blueprint, detailing how each could be applied across different phases to guide future airway teams.

**Conclusions:**

An implementation blueprint for a PED pre-intubation checklist was collaboratively developed with interprofessional AC team members and implementation scientists. This blueprint includes a manageable set of prioritized barriers and detailed strategies to navigate the implementation process. Future steps involve implementation of the checklist with concurrent evaluation of implementation and patient outcomes.

## BACKGROUND

Tracheal intubation (TI), a procedure in which an endotracheal tube is emergently placed for critically ill children with respiratory failure, is lifesaving yet high risk for adverse events. Multidisciplinary Pediatric Emergency Department (PED) clinicians must act quickly with limited patient information, minimal time for preparation, and while working within a rapidly-assembled ad hoc team. Adverse Airway Outcomes (AAOs) such as severe oxygen desaturation, hypotension, or cardiac arrest, occur in approximately 15–30% of PED intubations. Further, nearly one-third of cases require more than one attempt to properly place an endotracheal tube, and an increased number of attempts is associated with higher odds of adverse events [1–5]. Deviations from best practices are common and linked to increased procedural difficulty, team stress, mental fatigue, and poor clinical outcomes [3, 6, 7].

In pediatric and neonatal intensive care units, the use of checklists during TIs has been shown to reduce adverse events and improve outcomes in both single and multi-center settings [8–10]. However, compared to intubations that occur in intensive care units, TIs in the PED setting present unique challenges. Children requiring emergent airway management often arrive critically ill, unknown to the care team, and in acute distress leaving limited time to assess their clinical history or prepare for the procedure[1]. Further, TIs are performed less frequently in the PED setting compared to those in pediatric and neonatal ICUs [8, 11, 12]. Finally, the overall census and acuity of PEDs change rapidly, which may create resource challenges at the time of tracheal intubation. While checklists have been shown to improve intubation outcomes in general EDs [13, 14], these efforts have largely focused on adults and have been limited to a single institution. Given these challenges, the lack of a rigorously developed or widely adopted checklist for use in pediatric intubations in the PED setting is a critical opportunity to standardize pre-intubation processes and patient safety.

### NEAR4PEM Pre-Intubation Checklist

**Figure 1:**
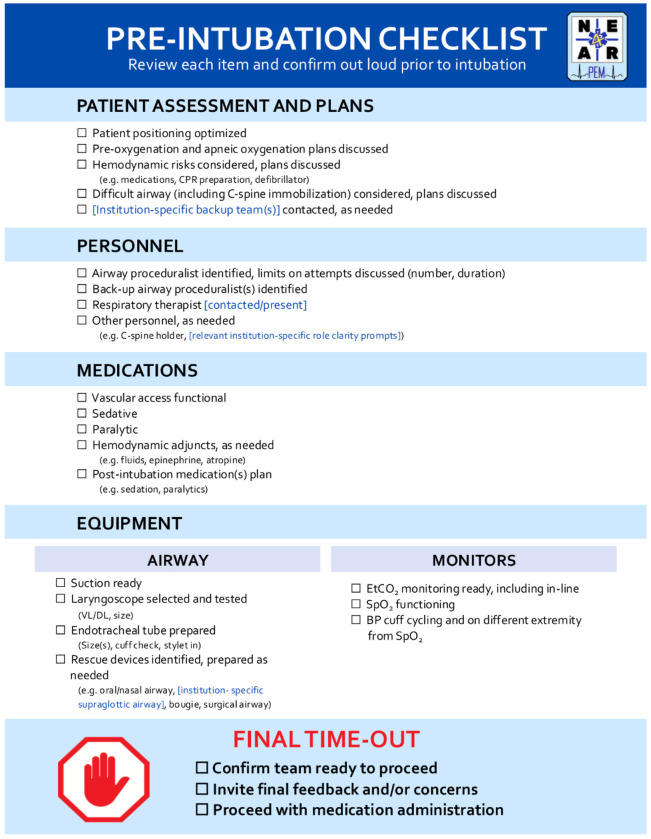
The NEAR4PEM Pre-Intubation Checklist

The National Emergency Airway Registry for Pediatric Emergency Medicine (NEAR4PEM) is a multicenter collaborative registry for advanced airway management in the PED [15, 16]. To address the unique challenges of pediatric TIs in the PED, NEAR4PEM developed a consensus-driven pre-intubation checklist using a multifaceted, rigorous approach. The development process included: 1) a modified Delphi approach to select checklist items [17], 2) focus groups led by human factors engineering experts to optimize the checklist for clinical use, and 3) usability testing and validation via high-fidelity simulation to further refine the checklist for use in the clinical setting [18]. The NEAR4PEM pre-intubation checklist (Fig. 1) was iteratively designed to align with clinicians’ cognitive and physical workflows in the PED setting. It balances comprehensiveness and utility while not being overly prescriptive or time-intensive. It is a 24-item checklist designed to be read aloud by the team leader immediately prior to intubations in the PED to facilitate procedural preparation and improve safety.

### Importance

Using a pediatric intubation checklist is a change in routine care for many PEDs. As such, we anticipated barriers to implementation and sought to develop sound implementation strategies to support widespread uptake and limit variability in adoption and use. In a prior PICU study, effective tactics for airway bundle implementation success included interprofessional quality improvement team involvement, while ineffective tactics included physician-only rollouts, lack of interdisciplinary education, lack of feedback data to frontline clinicians, and misconception of the bundle as research instead of quality improvement [20]. Understanding and addressing these and other potential or previously unrecognized contextual factors is an essential step to effectively implementing this new practice within the PED setting.

Creation of an implementation blueprint, a structured guide that outlines the activities, timelines, roles, and resources needed to support the successful implementation of an intervention [21], will assist with large-scale implementation and dissemination across the NEAR4PEM collaborative, and eventually potentially more broadly.

### Objective

The objective of this study is to use a participatory five step process with key parties to create an implementation blueprint to optimize the uptake of the NEAR4PEM pre-intubation checklist in PEDs. By taking a participatory approach—co-designing with frontline clinicians and other key informants from inception through rollout—we will enhance contextual fit, ownership, and feasibility, increasing the likelihood that implementation is effective, useful, and sustainable in real-world settings [22].

## METHODS

This was a prospective, mixed methods study performed prior to the implementation of the NEAR4PEM pre-intubation checklist in PEDs. Institutional Review Board (IRB) approval as exempt status (IRB #1978537-6) was obtained at the lead site (Lifespan/Brown University).

### Participants

The study cohort was composed of three groups of key informants: (1) Airway Champion (AC) teams, (2) a multi-site multidisciplinary Advisory Board, and (3) an Implementation Planning Team (IPT).

#### Airway Champion (AC) teams.

NEAR4PEM physician site leads identified frontline clinician key informants at their institutions —clinicians knowledgeable about and invested in pediatric ED airway management—to form local quality-improvement AC teams. Teams included PED physicians (including site PIs), pediatric trauma team physicians, nurses, respiratory therapists, and pharmacists. Minimum requirements were at least one physician, one nurse, and one respiratory therapist per team. AC team members were invited by email to participate in the survey (Step 1) and were also invited to join the Advisory Board.

#### Advisory Board (AB).

The Advisory Board was composed of multidisciplinary and interprofessional AC representatives purposefully sampled to represent varying annual PED visit volume, geographic location, and prior checklist experience level, as well as a PICU physician who had prior experience with the implementation of an intubation checklist in the PICU. The AB reviewed quantitative and qualitative survey findings, provided input on strategy prioritization (Step 4) and translated findings into an implementation blueprint (Step 5).

#### Implementation Planning Team (IPT).

The IPT consisted of implementation science experts and a subset of AB members. The IPT led strategy selection (Step 3).

### Procedures

**Figure 2:**
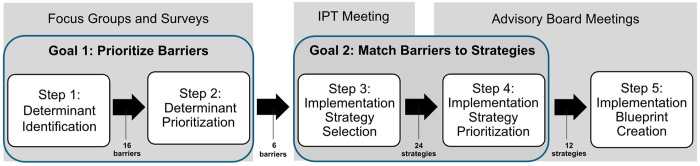
Methodology for Implementation Blueprint Creation This figure demonstrates the five steps of tailoring methodology, grouped by goals and team meetings with resulting outputs (further described in Results). IPT: Implementation Planning Team

Our methodology consisted of a modified conjoint analysis, modeled after the Lewis method [21]. Our five steps were: (1) formative evaluation for determinant identification, (2) determinant prioritization (focusing on barriers), (3) implementation strategy selection, (4) implementation strategy prioritization, and (5) implementation blueprint creation. (Fig. 2) Conjoint analysis [23] is a method that helps key informants evaluate and prioritize product features, services, or strategies by assigning value to different attributes to inform ultimate product design, or in this case, the implementation strategies included in the implementation blueprint. This analysis supports engagement of key informants in the clarification and prioritization of barriers and selecting strategies to enhance implementation. Key informant input is gathered through rating or sorting tasks, such as ranking feasibility and acceptability.

#### Step 1: Formative evaluation to identify determinants of checklist implementation

To systematically identify determinants to checklist implementation, we employed a two-phase, exploratory sequential mixed-methods approach, in which qualitative findings informed items included in a quantitative survey [24]. In the first phase, we performed focus groups guided by the Consolidated Framework for Implementation Research (CFIR 2.0) [25] with multidisciplinary key informants to assess and identify determinants of implementing the NEAR4PEM Pre-Intubation Checklist in PEDs. Full methods and results for this phase have been presented separately [26]. Briefiy, focus groups composed of physicians, nurses, pharmacists, and respiratory therapists were conducted at four NEAR4PEM sites purposefully sampled to represent varying annual visit volume, geographic location, and prior checklist experience. Barriers and facilitators were coded using CFIR domains and constructs and grouped into clinically relevant themes using a framework matrix approach [27]. Nineteen key informants from 4 hospital systems noted facilitators and barriers across all CFIR five domains.

#### Step 2: Prioritizing determinants to target with implementation strategies using modified conjoint analysis

Findings from the focus groups were used to develop a quantitative structured survey to identify barriers to checklist use in PEDs and to rank the importance and feasibility of addressing each barrier in participant’s clinical settings. This survey was piloted with local, interprofessional clinicians, whose feedback was integrated to improve readability, clarity, and acceptability [28, 29]. It was then distributed to all NEAR4PEM site ACs to ensure representation of site-level variability and enhance the generalizability and validity of the identified barriers. To develop the strategies for the implementation blueprint, we focused on identifying and clarifying barriers. Facilitators were also documented with the intention of leveraging them in the implementation plan. We also requested demographic information and perception of checklist impact on clinical outcomes. (Additional File 1).

Developing implementation strategies to address the most “high-priority” barriers enables efficiency with implementation time, energy, and resources [30]. A barrier may be designated “high priority” if it is both important and feasible to address [31]. To prioritize barriers, we asked participants to rate each identified barrier’s relative importance (i.e., high or low impact on checklist implementation) and the feasibility of addressing the barrier (i.e., high or low perceived ability to change the barrier) with a 4-point Likert scale. See “Statistical Analysis” section below for detailed statistical analysis.

#### Steps 3 and 4: Implementation strategy selection and prioritization in partnership with an Advisory Board

The Implementation Planning Team (IPT) met virtually to identify implementation strategies that conceptually matched the set of prioritized barriers using the Expert Recommendations for Implementing Change (ERIC) taxonomy [32]. This team was comprised of 11 team members including implementation science experts (n = 2), NEAR4PEM physician site PIs (n = 6), a respiratory therapist, and PICU physicians who had prior experience with implementation of an intubation checklist in the PICU (n = 2). The initial portion of the meeting involved providing an overview of the survey results with prioritized barriers. We then familiarized participants with the ERIC taxonomy, including strategies and definitions. Facilitators (RW and AA) led the group in a discussion to select appropriate ERIC strategies for each of the top priority barriers, including discussing each strategy as potentially relevant to the checklist implementation context. Specifically, for each priority barrier, attendees reviewed the provided ERIC strategy list and, drawing on their clinical experience, identified candidate strategies. They then discussed which options were most appropriate and why. To ensure that relevant strategies were not overlooked, we also used the CFIR-ERIC Implementation Strategy Matching Tool as an additional guide [33].

The selected ERIC strategies and definitions were then presented to the Advisory Board during a virtual meeting. The Advisory Board was composed of 6 PEM physicians, 2 respiratory therapists, 1 PEM nurse, 2 PEM Trauma team representatives, and a PICU physician who had prior experience with implementation of an intubation checklist in the PICU. Board members were divided into five interprofessional, cross site breakout groups, each assigned five strategies to evaluate. Group members discussed and ranked strategy feasibility (i.e. “high” or “low” feasibility of the strategy) and impact (i.e. “high”, “moderate” or “low” impact of the strategy on checklist implementation) based on their current clinical setting and resources on an open virtual board (Lucid Chart). A facilitator (RW) then led an intergroup discussion during which each group presented their rankings and any suggested modifications were reviewed. Strategies were selected for inclusion in the blueprint if they were rated as high/moderate impact and high feasibility.

#### Step 5: Implementation blueprint creation to operationalize all strategies in line with reporting guidelines

Guided by the Rudd pragmatic implementation strategy reporting tool [34], we then organized the top-ranking strategies into an implementation blueprint. This tool combines the ERIC taxonomy [32] with Proctor guidelines [35] for implementation strategy reporting. It importantly prompts consideration of a detailed operationalization of each implementation strategy including action targets, timing, and dose. We expanded this tool to incorporate necessary implementation materials aligned with each strategy’s goal. The draft blueprint and associated materials were then reviewed by Advisory Board members in subsequent meetings and refined to ensure that strategies were complete.

### Statistical Analysis

We described the participants by the count and proportions in %. To analyze the results for feasibility and impact to address barriers, we summarized the data based on the means, standard deviations, and correlation in responses as implied by a bivariate normal distribution [36].

For Step 2, means and correlations between responses to the same barriers were modeled based on the normal distribution. We considered those barriers with the highest average ratings of feasibility and impact the most actionable. Additionally, we anticipated that an actionable barrier would not just have high average ratings of feasibility and impact, but also low variance in ratings, and minimal correlation between ratings of feasibility and impact. This would indicate that there was agreement on the potential for addressing the barrier. This can be taken in contrast to a barrier with higher variance and a negative correlation between ratings of feasibility and impact. This would represent a controversial barrier: i.e., some believing it is feasible but not impactful, others thinking it would be impactful but not feasible. To further guide interpretation, we performed a cluster analysis with a dendrogram on the responses to each barrier. This approach analytically identified barriers with similar response patterns, which helped guide us toward barriers that demonstrated the pattern of responses we thought would represent an actionable barrier. Additional details on the statistical approach for survey results in Step 2 are provided in Additional File 2.

## RESULTS

### Steps 1 and 2: Formative evaluation for determinant identification and determinant prioritization

In focus groups, nineteen key informants from 4 hospital systems noted facilitators and barriers across CFIR domains [26]. A total of 16 unique barriers were identified, with the most prominent including high staff turnover, team resistance to change, and perceived lack of need for a checklist. Additionally, 19 facilitators were identified, with key facilitators including adequate staff training, communication and delivery to all key informants, and adaptability of the checklist.

Survey participants included 45 AC team members from 13 NEAR4PEM sites (12 sites were Level 1 trauma centers). Key informants included PEM physicians (n = 24), PEM nurses (n = 9), respiratory therapists (n = 8), trauma team members (n = 4), and pharmacists (n = 3). Three participants identified themselves as more than one role.

**Figure 3:**
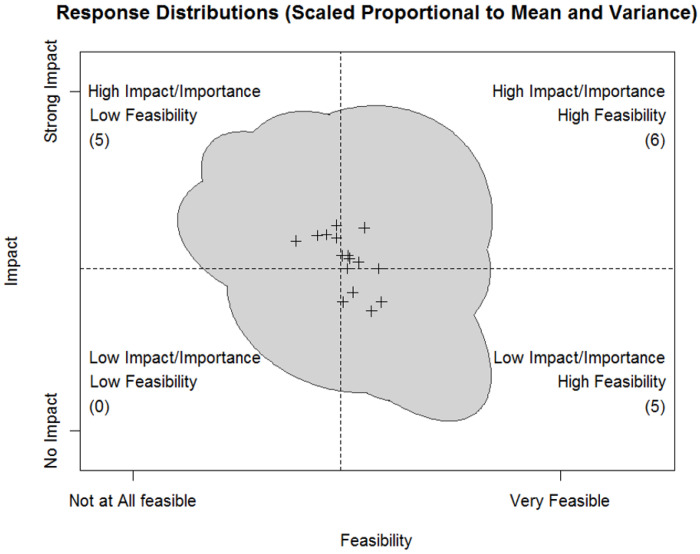
Relative Distribution of Prioritized Barriers Plus signs represent average common values for each barrier, The grey shaded area represents where 90% of response ratings were based on the bivariate distribution of rating impact and feasibility. Two barriers had means on the border between the top right (high impact/importance, high feasibility) and bottom right (low impact/importance, high feasibility) quadrants. Aware that Likert scales are inherently arbitrary, the responses were scaled to the grand mean and overall variance for all barrier ratings of feasibility and impact. This helped us distinguish relatively higher and lower ratings of feasibility and impact contextualized by the response categories. Decisions on how to classify these were based on the cluster analysis results, which better accounted for the distribution of individual ratings beyond the average alone; these skewed upper right for one barrier and lower right for the other.

Six of the 16 unique barriers were prioritized by the conjoint analysis (i.e. rated as high feasibility and high impact) (Fig. 3 and Table 2). Across ratings, feasibility received consistently higher endorsements than impact. Because of this, impact and feasibility ratings were interpreted in comparison to the average rating of impact or feasibility across barriers, rather than in terms of the original scaling from 1 (low impact/feasibility) to 4 (high impact/feasibility). This approach acknowledged that participants clearly thought the larger challenge is impact but still allowed for an interpretation of which barriers could have relatively greater impact across barriers.

### Step 3: Implementation strategy selection

The Implementation Planning Team collaboratively selected 37 potential implementation strategies for the set of prioritized barriers. The CFIR-ERIC Implementation Strategy Matching Tool [33] provided no additional strategies. After the strategy selection meeting, the study PI and IS consultant (RW, AA) reviewed the selected strategies to combine like strategies and remove a priori strategies. This resulted in 24 unique strategies from the session.

### Step 4: Implementation strategy prioritization and Step 5: Implementation blueprint creation

The Advisory Board ranked 19 ERIC strategies from 5 ERIC categories as high/moderate impact and high feasibility to address the prioritized barriers. Top-rated strategies included audit and feedback, promoting adaptability, and facilitating relay of clinical data to providers. Finally, an implementation blueprint was created which detailed the operationalization of top-rated strategies (Table 3). Strategies appear in chronological order by implementation phase and dose (gray headings). Of note, a priori strategies were included in the blueprint though were not ranked.

## DISCUSSION

Despite the high-risk nature of emergent pediatric intubation and supportive data for procedural checklists elsewhere, research on checklist use and implementation in the PED is still sparse. This study utilized a mixed methods, theory driven, participatory approach for developing an implementation blueprint to guide the incorporation of the NEAR4PEM pre-intubation checklist in clinical practice. By leveraging key informants to identify, specify, and prioritize multidisciplinary, multicomponent barriers, we matched implementation strategies to the CFIR determinants perceived to be most responsible for Checklist use. This determinant-strategy mapping (with explicit actors, actions, dose, and timing) yields a pragmatic, testable blueprint tailored to complex, dynamic conditions in the PED. Such tailoring increases the likelihood of successful implementation, higher-fidelity use, and long-term sustainment.

Participatory approaches—spanning key informant engagement, co-design, and community-based participatory research—consistently improve contextual fit, adoption, and sustainability of implementation efforts and are central to advancing equity, making them well-aligned with our strategy selection and blueprint development [22, 37, 38]. However, participatory work in the PED is uniquely challenging: the environment is high-acuity and shift-based with little down time; team composition changes as other disciplines (e.g., anesthesia, surgery, respiratory therapy) cycle in and out based on patient needs; no two PEDs are alike, with substantial variation in volume, staffing models, trainees, and culture. Accordingly, our blueprint engagement plan emphasizes robust pre-implementation work, brief, frequent touchpoints, asynchronous feedback channels, and site-specific adaptation while preserving checklist core components.

Barrier prioritization showed that the six highest-priority items (high feasibility/high impact) clustered within the CFIR domains of Individuals, Inner Setting, and Process; none arose from Innovation or Outer Setting. Several ‘Individuals’ barriers were high-feasibility but low-impact. The lowest feasibility barrier was delivering education/training to subspecialty teams (e.g., otolaryngology, anesthesia, PICU, NICU) that are infrequently called to assist with intubation in the PED. Because their involvement is episodic and for the highest-risk airways, preparedness demands deliberate coordination—and checklist use may be especially beneficial in these cases to clarify roles and streamline equipment checks. Importantly, subspeciality team involvement frequency varies markedly across sites—some PEDs call anesthesia once a year, others weekly—driven by intubation volume, patient mix, and unit culture [39–42]. Accordingly, although not ranked among the top priorities overall, this barrier warrants site-specific strategies to maintain readiness for rare but high-stakes events.

In developing our implementation blueprint, we anchored strategy selection in the ERIC taxonomy and the bundle-implementation literature in acute care. In a scoping review of care bundle implementation in acute care settings, Gilhooly et al. [43] found studies used 1–13 strategies (median = 5) and collectively drew on 48 of the 73 ERIC strategies, most often advisory boards, ongoing training/educational meetings, and audit-and-feedback. By contrast, we specified 19 strategies, intentionally pairing education with evaluative/iterative methods (audit/feedback) and stakeholder-relationship building (airway champions, multidisciplinary teams). Our blueprint emphasizes strategies seen in Gilhooly’s high compliance sites - champions, multidisciplinary engagement, and formative evaluation – and avoids over-reliance on strategies seen in low compliance sites, such as reminders alone (posters/screensavers). We also include strategies not present in Gilhooly’s sample—conduct local consensus discussions, promote network weaving, and distribute educational materials—to improve local fit and cross-site spread. Their observation that fewer bundle elements enhance compliance supports our focus on a concise, high-yield checklist while tailoring implementation, not the clinical content, to context. Finally, echoing their call for standardized reporting of implementation strategies, we specify ERIC names and Proctor parameters (actor, action, dose, timing, targets) to enable reproducibility and fidelity monitoring [32, 35].

Our blueprint also builds on well-accepted ED implementation-science examples—Li et al. 2021 and Southerland et al. 2023—while adapting strategy emphasis to the emergent, procedure-focused context of pediatric intubation [44, 45]. Like Li’s syncope Clinical Practice Guideline work, our blueprint highlights identifying and preparing champions, developing educational materials, educational meetings, and dynamic training. However, Li also selected outer-setting, patient-engagement strategies (e.g., preparing patients to be active participants, involving family caregivers, and equipping clinicians with communication tools). These strategies are less applicable to our intervention when the immediate goal is a safe, time-critical procedure in a distressed child. In contrast, Southerland’s CFIR-guided geriatric screening highlights inner-setting realities highly relevant to our context—unit/shift cultural heterogeneity, staff turnover, and the value of team-level audit/feedback. These insights reinforce our emphasis on inner-setting/process directed strategies (e.g., champions, iterative audit/feedback, on-shift education) and our addition of consensus discussion and network weaving to accelerate cross-site learning, while deemphasizing patient-facing strategies that do not map cleanly to emergent intubations.

Although developed for pediatric ED intubation, our mapping of implementation determinants to implementation strategies and operationalization may be applicable to other time-critical ED interventions (e.g., sepsis bundles, asthma pathways, procedural sedation). Because the blueprint specifies actors, actions, dose, and timing (Proctor parameters), teams can ‘swap the target behavior’ while retaining core strategies—local champions, dynamic training, embedded workflow supports, audit/feedback, network-weaving, and tailored data relay—then adapt to local inner-setting nuances (staffing models, consultant involvement, volume, unit culture). This makes the blueprint a reusable, transparent starting point for designing, reporting, and iterating implementation plans across heterogeneous ED settings, especially for low-frequency/high-stakes workflows.

### Limitations

These findings must be considered in light of inherent methodological limitations. While we tried to maximize generalizability of our results by recruiting multiple institutions from different geographical areas, participants at each institution were self-selected samples which raises concern for selection bias and may affect generalizability. Our barrier identification/prioritization relied on site PIs/Airway Champions; some disciplines (e.g., nursing, RT, anesthesia, ENT) were under-represented. However, Huntink et al [46] found little to no difference in strategy generation across key informant categories (e.g., researchers, quality officers, health professionals), suggesting that involvement of key informants is important but equal representation and contribution are not necessary for sound strategy selection. Lastly, “impact” and “feasibility” ratings reflect beliefs, not observed effects, so they may not predict what determines implementation outcomes.

## CONCLUSIONS

This collaboratively developed blueprint for implementation of a quality improvement tool for EDs includes a manageable set of prioritized barriers and a clear plan for which strategies to engage by whom and when in the implementation process. Next steps involve a blueprint driven pilot implementation of the NEAR4PEM pre-intubation checklist while assessing implementation outcomes (reach, adoption, fidelity, feasibility, acceptability). The study findings from this pilot will directly feed into a larger-scale checklist rollout as a multi-site Type III hybrid effectiveness-implementation trial, testing both effectiveness and implementation strategies across diverse PEDs. Future adaptations may be made for use of the checklist and blueprint in the broader ED settings.

## Supplementary Material

This is a list of supplementary files associated with this preprint. Click to download.

• AdditionalFile1.NEAR4PEMChecklistDeterminantSurvey.docx

• AdditionalFile2.StatisticalAnalysis.docx

## Figures and Tables

**Table 1:** Survey (Steps 1 and 2) Participant Characteristics

	n (%)
NEAR4PEM AC Team Role:	
PI or Co-PI Role (%)	17 (32%)
Nurse Champion Role (%)	9 (21%)
RT Champion Role (%)	8 (17%)
Physician (Non-PI) Role (%)	7 (15%)
Trauma Champion Role (%)	4 (7%)
Pharmacist Role (%)	3 (6%)
Female Gender (%)	30 (62%)
Use Pre-Intubation Checklist (%)	27 (57%)
Site Trauma Level*	
Level 1	41 (91%)
Level 2	1 (2%)
Level 3	3 (7%)
	**Median (IQR)**
Age (Years)	42 (36, 47)
Experience in PED (Years)	11 (7, 18)
Experience at Current PED (Years)	8 (4, 17)

AC: Airway Champion, PED: Pediatric Emergency Department

* Site Trauma Levels refer to hospital capability (I = highest resources with 24/7 in-house trauma surgeon & subspecialists; II = similar without research volume; III = stabilize, emergency operations with transfer as needed; IV/V = initial stabilization + transfer)

**Table 2:** Barrier Ratings

		Mean (SD)		
Barrier	CFIR Domain	Impact	Feasibility	Correlation
High Impact, High Feasibility				
Team members will not know who should lead the Checklist during an intubation	Individual	2.74 (0.82)	3.33 (0.68)	0.09
Team will have difficulty consistently accessing/locating the Checklist for use during resuscitations	Inner Setting	2.38 (0.77)	3.29 (0.64)	−0.23
We will have difficulty providing training/education due to other competing job responsibilities	Inner Setting	2.45 (0.6)	3.2 (0.65)	−0.42
Team members will lose interest in Checklist utilization if they do not feel included in the implementation process and/or are not informed about outcomes. (Lack of feedback incorporation about checklist and checklist use)	Process	2.42 (0.62)	3.21 (0.53)	−0.43
We will have difficulty providing education/training about the Checklist (for an infrequent procedure) to a large number of staff due to high rate of staff attrition and onboarding (high staff turnover)	Process	2.45 (0.51)	3.15 (0.65)	−0.71
Multidisciplinary and interdepartmental groups have not traditionally been involved in quality improvement initiatives	Process	2.32 (0.74)	3.2 (0.59)	−0.37
High Feasibility, Lower Impact				
Team members are unaware, or fail to acknowledge, that emergent pediatric intubation is a procedure with many risks	Individual	1.97 (0.8)	3.47 (0.58)	−0.62
Divisional/department leadership does not/will not support use of the Checklist	Individual	1.88 (0.69)	3.39 (0.53)	−0.77
Team members are unaware, or fail to acknowledge, that checklist use has improved outcomes for pediatric intubation in the PICU and NICU settings	Individual	2.32 (0.58)	3.45 (0.6)	−0.02
Our team is just not familiar with utilizing checklists in the clinical setting (Lack of ‘checklist culture’)	Inner Setting	2.07 (0.76)	3.24 (0.62)	−0.55
Team members may fear legal risks created by this quality improvement initiative (e.g. negative consequences if not followed precisely)	Outer Setting	1.97 (0.61)	3.16 (0.62)	−0.18
High Impact, Lower Feasibility				
We will have difficulty providing education/training to staff in ancillary departments that are more rarely involved in pediatric intubation in the PED (e.g. ENT, anesthesia, PICU, NICU)	Inner Setting	2.6 (0.49)	2.78 (0.63)	−0.29
Team Members feel that they do not need a checklist because they already know the steps and equipment necessary for intubation	Individual	2.76 (0.77)	3.11 (0.55)	−0.32
We have difficulty continuing use of quality initiatives after their initial introduction due to a lack of process for sustainment	Process	2.66 (0.7)	2.95 (0.62)	−0.64
Team members will be unwilling to adapt to using the Checklist and will continue current intubation workflow	Inner Setting	2.67 (0.56)	3.02 (0.51)	−0.74
Team members perceive that the Checklist may take too long to complete	Innovation	2.63 (0.71)	3.11 (0.67)	−0.37

**Table 3:** NEAR4PEM Pre-Intubation Checklist Implementation Blueprint

Barriers	ERIC Strategy	Operationalization	Justification	Related Resource
Pre-Implementation				
n/a	Develop a formal implementation blueprint^a^	Create formal implementation blueprint based on input from AB	To operationalize strategies into a format that can guide implementation and enable tracking and replication.	Blueprint
n/a	Identify and prepare champions^b^	Identify multidisciplinary Airway Champion (AC) teams at each site and specify their role with Checklist Implementation	Multidisciplinary leadership and endorsement are critical to foster collective ownership and drive adoption of the checklist across all clinicians involved in emergent airway management.	
Lack of interdepartmental/interdisciplinary input in prior QI	Obtain formal commitments	Obtain formal commitments from site PI and site leadership outlining participation requirements and commitment to checklist implementation and sustainability.	Strong leadership endorsement, coupled with explicit clarification of roles and responsibilities, will foster shared understanding and promote consistent checklist use.	Endorsement Letter
Checklist leader High staff turnover Competing job responsibilities	Develop educational materials	Develop accessible educational materials (instruction guide, educational video, slides, simulation cases) to orient teams to checklist use (including leader).	Accessible, standardized education clarifies leadership roles for Checklist use and provides consistent training that can be reused for new staff and delivered flexibly to accommodate high turnover and competing clinical demands.	Educational Video, Slide Deck, Simulation Guide
High staff turnover Competing job responsibilities	Make training dynamic	Offer varied training through multiple formats—including short videos, Q&A, in-person didactics, and simulation cases—so that education can be accessed flexibly across different work contexts and schedules.	Dynamic training, particularly asynchronous learning components, ensures consistent onboarding despite staff turnover, accommodates competing job responsibilities, and engages different learner types through interactive and varied methods.	Educational Video, Slide Deck, Simulation Guide
High staff turnover	Use train-the-trainer strategies	Site PIs and ACs complete all educational materials and participate in focused meetings to ensure mastery of checklist use. Training emphasizes strategies to engage learners, incentivize module completion, and address questions about checklist application.	Champions are equipped to lead educational efforts at intervals dictated by statt turnover, which can be unpredictable, ensuring consistent onboarding and reinforcement. Enabling more team members to be trainers allows for better preservation of institutional knowledge despite high turnover.	Check In Guide
Lack of interdepartmental/interdisciplinary input in prior QI	Promote network weaving	Build on existing high-quality site-level multidisciplinary teams (such as hospital-wide airway teams and/or Quality Improvement committees) to promote information sharing and QI involvement and expand shared vision for emergent airway management.	Within sites, network weaving across multidisciplinary units will strengthen collaboration and promote shared ownership of the checklist.	Check In Guide
Lack of interdepartmental/interdisciplinary input in prior QI High staff turnover	Create a learning collaborative^b^	Create a learning collaborative of multidisciplinary ACs to share knowledge and experience about checklist use and training approaches.	Recruiting an airway champion from each discipline ensures meaningful involvement in the QI initiative and leverages their insight into staff turnover patterns, enabling more effective and timely education of new team members. AC members can also share tools and effective training practices.	
Pre-Implementation AND during Implementation Phase as needed				
Checklist location	Promote adaptability	Checklist placement will be selected to optimize accessibility in each site’s unique clinical environment, with locations modified if barriers are identified.	If clinicians cannot find the checklist quickly in a high-stress environment, they are unlikely to use it. PEDs can change rapidly, so the optimal checklist location may change over time.	Check In Guide
Checklist leader Checklist location	Model and simulate change	Develop and facilitate multidisciplinary simulations (ideally in situ) to practice checklist use during clinical care of patients with acute respiratory failure	Practice with the checklist in the clinical setting will not only build ease of use during patient care but also help sites identify the most effective checklist leader and refine checklist placement for optimal accessibility.	Simulation Guide
Checklist leader High staff turnover	Conduct educational meetings	Site PIs to conduct educational meetings of different key informant groups to enhance education/training about checklist at their site.	Educational meetings will ensure all team members understand checklist content, roles (including leader), and workflow, supporting consistent and proper use.	Educational Video and Slide Deck
Checklist leader High staff turnover Competing job responsibilities	Distribute educational materials	Distribute educational materials to all key informants to orient teams to checklist use (including leader) through pre-existing division newsletters, meetings/conferences, and educational sessions.	Through creating concerted and varied dissemination channels we will ensure that the education reaches the clinical staff, despite challenges from high staff turnover and competing job responsibilities	Instruction Manual
Regular Study PI/Site PI Meetings (weekly to monthly once implementation established)				
Checklist leader Checklist location	Audit and provide feedback	Checklist use will be reviewed by site PIs and study PI to identify issues and provide feedback to problem solve them.	To ensure that checklist is being used correctly and consistently with attention to leader and location.	Instruction Manual
Lack of feedback incorporation	Provide ongoing consultation	Study PI will provide guidance to Site PIs on how to relay implementation and clinical outcomes to the clinical teams; Encourage establishment of critical airway review teams at sites to critically review intubations.	Through ongoing consultation with study PI, sites will have support and guidance in keeping the teams informed about checklist performance.	Check In Guide
Monthly Site AC Meetings				
Checklist location	Purposely reexamine the implementation	AC teams will review feedback obtained from front line clinicians about whether checklist location is still easily accessible (physically able to access and where team members think to look for it) despite any physical or operational changes in the clinical setting.	Clinicians must be able to easily locate the checklist quickly in a rapidly changing chaotic clinical environment. This location may change over time so must be re-examined at regular intervals.	Check In Guide
Checklist leader Checklist location	Conduct local consensus discussions	AC teams will discuss optimal checklist leader and location based on potentially changing needs of the department and frontline clinician feedback.	Consensus discussions will inform decisions about checklist leader and location.	Check In Guide
Quarterly Multisite NEAR4PEM Study Team Meetings				
Checklist leader	Capture and share local knowledge	Obtain feedback from site PIs about optimal checklist leader at their sites and have them share this (and other implementation tips) at NEAR4PEM Quarterly meetings with other site PIs.	Through cross-site communication, site PIs can highlight which individuals or roles have proven most effective as checklist leaders within their institutions, providing practical guidance to inform leadership selection at other sites.	Check In Guide
Lack of interdepartmental/interdisciplinary input in prior QI	Promote network weaving	Build on existing high-quality multisite NEAR4PEM network to promote information sharing and shared vision for checklist use across disciplines, including shared engagement tactics and peer-troubleshooting of barriers.	Across sites, exchanging best practices will not only enhance optimal checklist use but also support sites less experienced with QI, helping them build confidence and capacity to engage in these activities.	Check In Guide
Regular Site-Specific Communications (Monthly to Quarterly, based on site volume)				
Lack of feedback incorporation	Facilitate relay of clinical data to providers	Facilitate relay of clinical data to providers by sharing implementation and clinical outcomes in a regular, timely fashion. Site PIs will distribute checklist use data to their staff using communication methods best suited to their unit (e.g., weekly division emails, staff huddles).	Relaying information and preserving space and resources for troubleshooting will help clinicians remain engaged and feel supported in the implementation effort.	Check In Guide
Lack of feedback incorporation	Remind clinicians	Site PIs to provide clinician reminders and reach out directly to team leaders if checklist is not used in an intubation to discuss barriers to use, opening up an opportunity for feedback from clinicians.	Providing reminders about checklist use gives clinicians opportunities to provide feedback, ask questions, and troubleshoot issues, while also helping them feel connected and engaged in the implementation effort.	Check In Guide

*Barrier* - prioritized barriers, identified by CFIR 2.0 qualitative focus groups and surveys, abbreviated for clarity (full barrier statements in Table 2); *ERIC Strategy* - Expert Recommendations for Implementing Change, all strategies originated during IS working group meetings unless otherwise noted; *Operationalization* - informed by Proctor framework for reporting strategies (Proctor, 2013); Related Resource - tools created to enact strategies.

a Preplanned Strategy,

b Preplanned strategy and from IS working group

*AC*: Airway Champions, *AB*: Advisory Board, *QI*: Quality Improvement

## Data Availability

Partial or complete deidentified datasets and data dictionary are available upon request to Dr. Wing at email rwing1@brownhealth.org to investigators who provide an IRB letter of approval.
